# Age‐Associated Gut Microbiota Dysbiosis Impairs Antiviral Defense During Influenza Virus Infection and Identifies Acetate as a Protective Mediator in Senescent Human Lung Cells

**DOI:** 10.1111/acel.70750

**Published:** 2026-10-07

**Authors:** Christoph Wernike, Antje Häder, Yasmina Reisser, Kiana Garakani, Franziska Hornung, Claude Jourdan Le Saux, Stefanie Deinhardt‐Emmer

**Affiliations:** ^1^ Institute of Medical Microbiology Jena University Hospital Jena Germany; ^2^ Else Kröner Graduate School for Medical Students “JSAM” Jena University Hospital Jena Germany; ^3^ Medicine/Pulmonary and Critical Care Division University of California San Francisco San Francisco California USA

**Keywords:** aging, gut microbiome, gut‐lung axis, influenza a virus, short‐chain fatty acids

## Abstract

Aging is associated with profound alterations in gut microbiota composition and function, including reduced production of short‐chain fatty acids (SCFAs), yet it remains unclear whether these age‐related microbial changes directly contribute to impaired antiviral defense during influenza A virus (IAV) infection. To address this question, we first characterized age‐associated alterations of the gut microbiome and SCFA‐related metabolic potential in a murine model of IAV infection. We then used a complementary human senescent lung fibroblast model to determine whether acetate, a major microbiota‐derived SCFA, directly modulates antiviral responses in aging‐associated lung cells. In old mice, IAV infection was associated with marked microbiota dysbiosis, including reduced abundance of SCFA‐producing commensals such as *Akkermansia muciniphila* and *Faecalibaculum rodentium*, accompanied by decreased expression of microbial pathways involved in carbohydrate fermentation and acetate production. These findings suggested impaired microbiota‐derived metabolic support of antiviral immunity during aging. Based on these observations, mechanistic studies in senescent human lung fibroblasts demonstrated that acetate supplementation significantly reduced viral replication and inflammatory responses. These protective effects were associated with FFAR2/FFAR3 signaling and enhanced histone H3 acetylation, indicating metabolic and epigenetic modulation of the antiviral response. Together, our findings establish a translational link between aging‐associated gut microbiota dysfunction and impaired antiviral defense by combining a murine model of aging and influenza infection with mechanistic validation in human senescent lung cells. They identify acetate as a potential mediator of the gut–lung axis and support microbiota‐targeted interventions to reduce influenza severity in older individuals.

## Graphical abstract

Aging altered the gut microbiota, reduced short‐chain fatty acid (SCFA) potential, and worsened influenza A virus (IAV)‐associated dysbiosis. In senescent human lung fibroblasts, acetate reduced IAV replication and inflammation via GPR41/43 signaling and histone acetylation, suggesting that SCFAs help protect antiviral defense along the gut–lung axis.

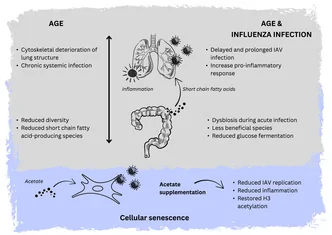

## 1 Introduction

Aging profoundly reshapes the composition and functional capacity of the gut microbiome, a complex community of microorganisms residing in the gastrointestinal tract. Throughout life, this microbial ecosystem supports digestion, contributes to host metabolism through the production of bioactive metabolites, and plays a central role in modulating immune function. With advancing age, the microbiome undergoes characteristic shifts in diversity and in the abundance of key functional bacterial groups, resulting in a state of dysbiosis. Such imbalances are associated with increased inflammation, compromised host resilience, and heightened susceptibility to disease, highlighting the critical impact of age‐related microbiome alterations on overall health (Bartley et al. 2017; DeJong et al. 2020; Hohman and Osborne 2022; Schoultz et al. 2025).

Studies in humans have shown that aging is both accompanied and influenced by shifts in the composition of the gut microbiota, particularly among functionally important bacterial groups and metabolic pathways (Woodmansey et al. 2004). For instance, age‐related changes often include a reduction in members of the phylum *Firmicutes* and *Bifidobacterium* and an increase in bacteria such as of the phylum *Bacteroidetes* and *Eubacterium* (Biagi et al. 2010; Claesson et al. 2011). At the species level, anaerobic bacteria are particularly susceptible to decline with age, including metabolically active species such as *Faecalibacterium prausnitzii* , members of *Actinobacteria*, and *Akkermansia muciniphila* (Biagi et al. 2010; Collado et al. 2007).

Research in humans and mice indicates that the gut microbiome not only influences local intestinal processes but also affects distant organs, including the respiratory system (Hoen et al. 2015; Hornung et al. 2025). Furthermore, Zhang et al. (2026) recently emphasized the gut as a central hub for multi‐organ communication in aging, highlighting its role as an integrative systemic regulator.

Moreover, respiratory infections can in turn impact gut microbial composition, even in the absence of detectable pathogens in the gastrointestinal tract (Groves et al. 2018). This bidirectional communication, mediated by microbial metabolites, immune cells, and inflammatory signals traveling between the gut and lungs, is referred to as the “gut–lung axis.” Disruption of this axis through microbial dysbiosis has been shown to compromise systemic immunity, thereby increasing susceptibility to respiratory infections such as influenza and COVID‐19 (Hohman and Osborne 2022; Hornung et al. 2025; Ichinohe et al. 2011; Pham et al. 2021).

The gut microbiome is a key producer of short‐chain fatty acids (SCFAs), including acetate, propionate, and butyrate, which are generated through the fermentation of dietary fibers. SCFAs support intestinal health and influence systemic physiology by modulating energy metabolism, immune responses, and inflammation. Following intestinal absorption, SCFAs can enter the systemic circulation and thereby provide a potential route for gut‐derived microbial metabolites to exert effects beyond the intestine (Shafaei et al. 2021). Through their immunomodulatory functions, SCFAs link microbial activity in the gut with systemic and pulmonary immune homeostasis, representing an important mechanism of gut–lung communication (Zhang et al. 2023). SCFAs represent an important mechanism of the gut–lung axis, linking microbial activity in the gut to immune function in the lungs (Zhang et al. 2023).

Emerging evidence indicates that SCFAs are important for maintaining lung health. For instance, butyrate acts as a histone deacetylase (HDAC) inhibitor, influencing gene expression to promote regulatory T cell differentiation and reduce pro‐inflammatory cytokine activity (Trompette et al. 2018). SCFAs also signal through G‐protein‐coupled receptors, GPR41 and GPR43, which regulate immune cell metabolism and dampen inflammatory responses in neutrophils and peripheral blood mononuclear cells (Cox et al. 2009).

Among SCFAs, acetate is particularly known to support antiviral defense (Antunes et al. 2022). Our own previous work has demonstrated that influenza A virus (IAV) exploits the SCFA receptor GPR43 to enter host cells. High extracellular acetate levels trigger translocation of GPR43 from the cell surface, reducing viral entry, whereas low acetate availability leaves receptors accessible, facilitating infection (Hornung et al. 2025). IAV serves as a relevant model for respiratory viral infections, as it is an enveloped, single‐stranded RNA virus of the *Orthomyxoviridae* family and a major cause of disease, particularly in older individuals (Paget et al. 2019).

At the cellular level, our previous work has demonstrated that human senescent cells are particularly susceptible to infection by IAV (Schulz et al. 2023). Senescent cells express a variety of cytokines, chemokines, proteases, and growth factors, which has been termed the senescence‐associated secretory phenotype (SASP) (Basisty et al. 2020; Coppé et al. 2008). These cells accumulate throughout the aging organism and can profoundly influence neighboring cells and tissue microenvironments, modulating local inflammation and immune responses.

Despite growing evidence that aging‐associated gut microbiota dysbiosis contributes to impaired immune function, it remains unclear whether age‐dependent alterations in microbiota‐derived metabolites directly influence antiviral defense in the aging lung. In particular, the mechanistic link between altered microbial metabolism during influenza infection and the increased susceptibility of senescent human lung cells has not been established.

To address this knowledge gap, we combined complementary in vivo and in vitro approaches. First, we characterized aging‐associated changes in gut microbiota composition and SCFA‐related metabolic pathways during influenza A virus infection in old and young mice to identify microbial mechanisms associated with impaired antiviral immunity. Based on these in vivo findings, we subsequently employed a human senescent lung fibroblast model to determine whether acetate, a major microbiota‐derived SCFA, directly modulates antiviral responses in aging‐associated lung cells.

By integrating physiological observations from a murine influenza model for aging with mechanistic studies in human senescent cells, our study provides a translational framework linking gut microbiota dysfunction, altered microbial metabolism, and cellular antiviral defense. These findings may help explain the increased severity of influenza in older individuals and identify microbiota‐derived metabolites as potential therapeutic targets to improve antiviral resilience during aging.

## 2 Results

### 2.1 Aging Drives Systemic Inflammation, Pulmonary Immune Activation, and Gut Microbiota Dysbiosis

To investigate the impact of aging on systemic and pulmonary physiology as well as gut microbial composition, we employed C57BL/6 mice at two distinct ages: young (8–12 weeks) and old (24 months) (Figure 1A).

**FIGURE 1:**
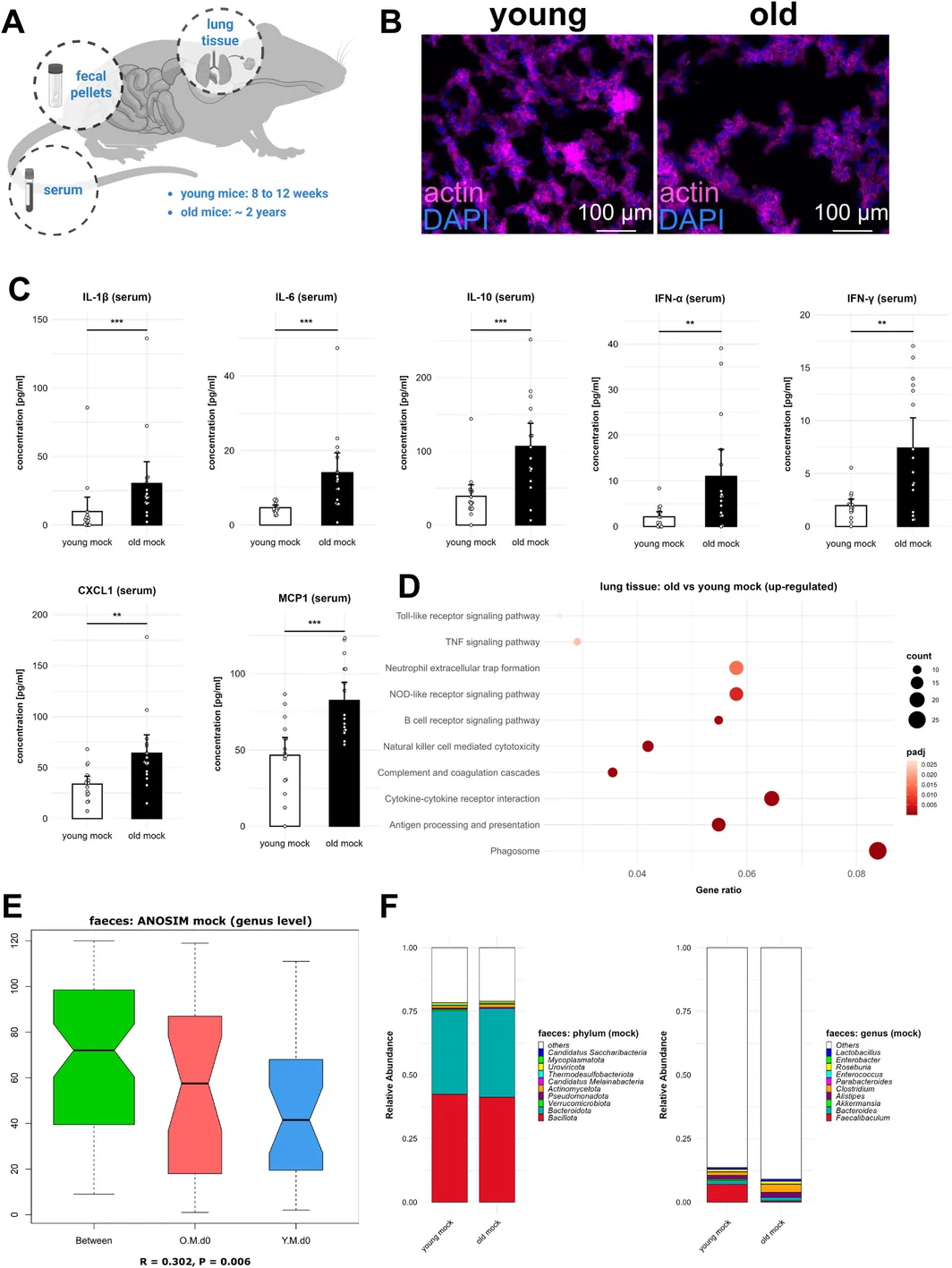
Aging is associated with changes in lung structure, chronic systemic inflammation and dysbiosis in old female C57Bl/6 mice compared to young specimens. (A) Schematic representation of the murine in vivo model of aging, consisting of a group of young mice (aged 8–12 weeks) and old mice (aged 2 years). (B) Changes in cytoskeletal structure of lung slices from old mice, with Phalloidin (purple) as a marker for Actin and DAPI as staining for nuclei. (C) Cytokine and chemokine levels in the serum of old mice compared to young (*n* = 16). (D) Enrichment analysis of upregulated KEGG pathways in the transcriptome of the lung tissue of old compared to young mice. (E) Analysis of similarities (ANOSIM) of gut microbial communities at the genus level in mock‐treated mice. Box plots show rank dissimilarities within groups (M.O., old mice; M.Y., young mice) and between groups. ANOSIM test indicating that similarities in the gut microbial composition on the genus level are greater inside the groups than between them. (F) Relative abundance of the 10 most abundant bacterial phyla and genera in the microbiota of young and old mice. **p < 0.01, ***p < 0.001. *p* calculated with Mann–Whitney‐Wilcoxon test.

We visualized nuclei and the actin cytoskeleton in murine lung sections to assess age‐associated structural alterations. Old lungs exhibited disrupted cytoskeletal architecture, reflecting structural deterioration compared to young tissue (Figure 1B). Old mice displayed pronounced systemic inflammation, with serum protein analysis revealing significantly elevated levels of interferons, pro‐inflammatory cytokines (IL‐6, IL‐1β) and chemokines (IL‐8, MCP‐1, CXCL‐1) compared to young mice (Figure 1C), consistent with chronic low‐grade inflammation (Davalos et al. 2010; Franceschi et al. 2000). Notably, the anti‐inflammatory cytokine IL‐10, a SASP factor implicated in immune suppression, was also elevated in the serum of old mice (Cao et al. 2025).

Transcriptomic profiling of lung tissue revealed substantial age‐dependent changes. Pathway analysis highlighted enrichment of inflammation‐related processes, including phagosome activity, antigen presentation, and cytokine‐cytokine receptor interaction, indicating heightened pulmonary immune activation in older mice (Figure 1D).

Gut microbiota composition also exhibited marked age‐associated shifts. Shotgun metagenomic analysis demonstrated an increased abundance of the genus *Clostridium* and a significant depletion of the genus *Faecalibaculum* in old mice (Figure 1E,F), reflecting a dysbiotic state that may contribute to systemic and pulmonary inflammation. Analysis of similarities (ANOSIM) revealed a significant difference between old and young mice in microbiota composition (Figure 1E).

Collectively, these findings reveal a coordinated profile of age‐associated alterations: systemic cytokine elevation, lung‐specific inflammatory transcriptomic changes, and gut microbiota dysbiosis. This integrated analysis of lung tissue highlights the interplay between aging, inflammation, and microbial community dynamics, providing a foundation for understanding the mechanisms underlying age‐related immune vulnerability.

### 2.2 Old Mice Exhibit Delayed but Exacerbated Immune Responses to IAV Infection

To assess the impact of aging on antiviral immunity, young (8–12 weeks) and old (24 months) C57BL/6 mice were infected intranasally with a sublethal dose of IAV (Figure 2A) (Hornung et al. 2023, 2025; Schulz et al. 2023). This influenza infection mouse model enables the analysis of both the acute phase and later recovery phases to capture not only early disease differences but also longer‐term host responses. Accordingly, we assessed Day 4 post infection as the acute phase, Day 8 as the post‐acute phase, and Day 21 as the long‐term/recovery phase, consistent with our previous influenza studies in high‐risk cohorts.

**FIGURE 2:**
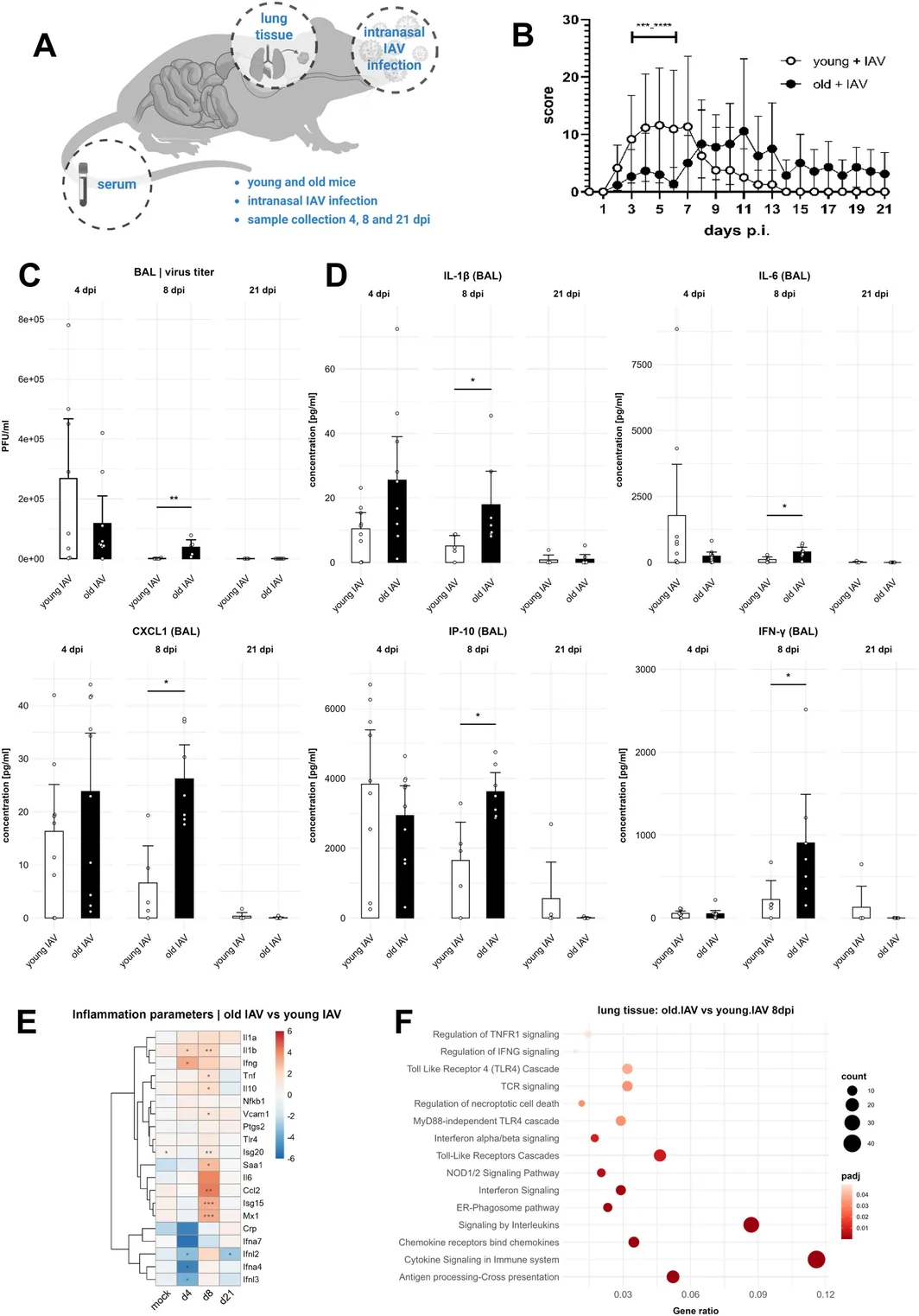
Influenza A virus (IAV) infection is associated with delayed and impaired immune response and prolonged inflammation in old mice. (A) Schematic design of IAV infection and sample collection. A total of 48 young and 48 old mice were included, comprising 10 infected and 6 mock‐treated animals per age group and time point (days 4, 8, and 21 post infection). The number of data points may vary between analyses, as animals reaching humane endpoint criteria prior to the planned experimental endpoint were excluded from specific downstream analyses. (B) The score was calculated for each day of infection based on clinical symptoms for each mouse (Table S1). (C) Quantification of the virus titer in BAL samples from infected mice at 4; 8 and 21 dpi via plaque assay. (D) Cytokine concentrations in the BAL of IAV‐infected and mock‐treated mice at 4; 8 and 21 dpi. (E) Heat map showing the gene expression log2foldchange of genes encoding inflammatory parameters in IAV‐infected old compared to young mice at 8 dpi. (F) Enrichment analysis of reactome pathways 8 dpi in the transcriptome of IAV‐infected old compared to young mice. *p < 0.05, ***p < 0.001, ****p < 0.0001. *p* calculated utilizing 2way ANOVA Šídák's multiple comparisons test (B), Mann–Whitney‐Wilcoxon test (C, D) and calculated with DESeq2 (E).

Distinct age‐dependent differences emerged in disease progression, viral load, and immune responses. Body weight differed significantly during the acute phase (Days 3–6 post infection) and, in the old cohort, did not fully recover by Day 21, remaining below baseline at the end of the observation period (Figure S1). To further capture parameters reflecting the course of infection in young and old mice, the clinical score of infected mice was assessed by observations and measurements as described in Table S1. Young mice displayed higher clinical scores during the early phase of infection (Figure 2B, Figure S1). In contrast, old mice showed a trend toward later clinical deterioration from day 8 onward, reflecting a delayed response. Correspondingly, BAL from old mice showed higher viral loads on day 8 (Figure 2C) and elevated levels of cytokines and chemokines, including IL‐6, IP‐10, and IFN‐γ (Figure 2D). Old mice showed already slightly enhanced IL‐1β and CXCL10 secretion in the uninfected state, but strongly increased levels due to IAV infection compared to young mice (Figure 2D, Figure S2).

Transcriptomic analysis of lung tissue mirrored these findings. On day 4 post‐infection, infected old mice exhibited modest inflammatory gene expression relative to infected young mice. Old mice showed relatively subdued gene expression changes associated with inflammation compared to young mice, with a significant decrease in expression of several Type I and Type III interferons (*Ifnl2, Ifna4, Ifnl3*). However, by day 8, old mice demonstrated a significant increase in the expression of inflammation‐related genes, aligning with the observed spike in cytokine and chemokine levels, with the exception of Type I and Type III interferons (Figure 2E, Figure S3). At this point in time, the global transcriptome response showed next to cytokine/chemokine signaling an enrichment of pattern recognition receptor signaling pathways, e.g., Toll‐like receptor cascades (Figure 2F, Figure S4).

These results demonstrate an age‐dependent response to IAV: young mice mount an early, transient response, whereas old mice show delayed viral clearance accompanied by exaggerated inflammation. This dysregulated immune response highlights how aging impairs early antiviral control and amplifies inflammatory pathology during respiratory viral infection.

### 2.3 IAV Infection Transiently Reshapes Gut Microbiome Composition and Metabolic Potential in Old Mice

To characterize the impact of aging and IAV infection on the gut microbiome, we performed shotgun metagenomic sequencing of fecal samples from young and old mice at baseline, Day 4 (acute phase), and Day 21 (chronic phase) post‐infection (Figure 3A).

**FIGURE 3:**
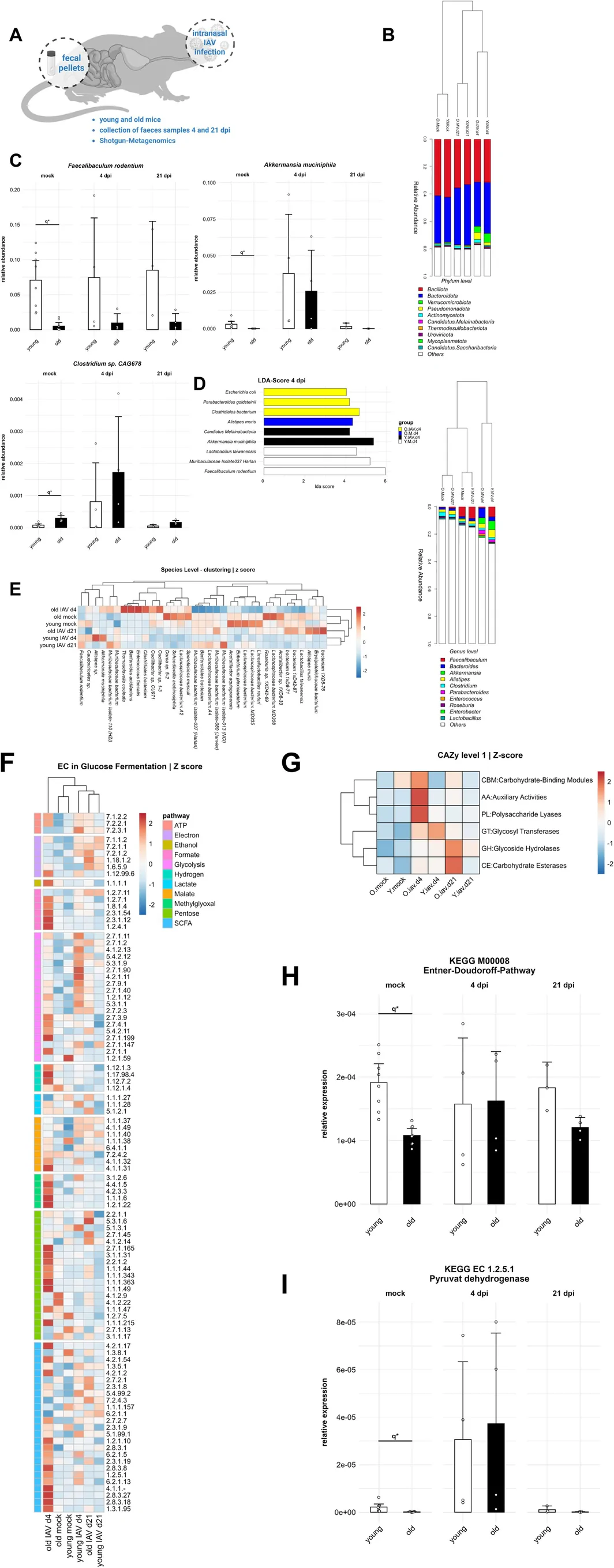
Old mice had a lower abundance of beneficial bacterial species, with an influenza A virus (IAV) infection leading to a further shift in composition and metabolism of the gut microbiome. (A) Feces were collected before the infection as well as 4 and 21 dpi and analyzed utilizing shotgun metagenomics. (B) Clustering of the relative abundance of the most abundant bacterial phyla and genera in the gut microbiota of IAV‐infected and mock‐treated old and young mice. (C) Abundance of *F. rodentium*, *A. muciniphila* , and *Clostridium* spp. in the gut microbiota of IAV‐infected and mock‐treated old and young mice. (D) LDA‐score showing the association of harmful and beneficial bacteria in the gut microbiota during the acute phase of IAV infection (4 dpi). (E) Species‐level heat map of the gut microbiota in young and old mice at 4 and 21 dpi, including mock‐treated controls. Values are *z*‐score transformed and clustered by hierarchical clustering. (F) Heat map of enzymes involved in glucose fermentation across gut microbiota samples from young and old mice at 4 and 21 dpi. Enzyme commission (EC) numbers are shown on the y‐axis, and *z*‐score‐transformed abundances are displayed by color. (G) Functional potential annotated to the CAZy database (a classification of carbohydrate‐active enzymes) revealed changes in carbohydrate metabolism at 4 and 21 dpi. The heat map shows *z*‐score‐transformed abundances of the indicated CAZy classes across the different sample groups. (H) Relative abundance of the Entner–Doudoroff pathway (glucose‐6P → glyceraldehyde‐3P + pyruvate; KEGG module M00008) in gut microbiota of young and old mice under mock conditions and at 4 and 21 days post infection. (I) Relative abundance of the enzyme pyruvate dehydrogenase (KEGG EC 1.2.5.1) in gut microbiota of young and old mice under mock conditions and at 4 and 21 days post infection. Pyruvate dehydrogenase (quinone) catalyzes the reaction from pyruvate to acetate. *q significant as calculated by Metastats analysis.

Our results revealed significant differences in the gut microbiome of old mice compared to young controls. As mentioned before, especially on the genus level a shift in composition was noticeable even before infection (Figure 1F). In the acute phase of the IAV infection, the composition of the gut microbiome is changing even further, with a decrease of *p. Bacillota* and increases in *pp. Verrucomicrobiota* and *Pseudomonadota* in both age groups. On the genus level, *g. Akkermansia* increases in both groups, most prominently however in the old mice (relative abundance_young_ = 0.064227; relative abundance_old_ = 0.043759). Further increases in relative abundance are noticeable in the *gg. Alistipes* (relative abundance_young_ = 0.055655; relative abundance_old_ = 0.030809) and *Parabacteroides* (relative abundance_young_ = 0.016639; relative abundance_old_ = 0.027781), the latter more prominently in old mice (lower panel of Figure 3B). On both phylum and genus level, data of bacterial relative abundance on Day 4 post‐infection cluster separately, stressing the impact of acute IAV infection on the gut microbiome composition. By Day 21 post‐infection, gut microbiome composition in both age groups showed a partial shift toward pre‐infection profiles, although infection‐associated alterations remained detectable (Figure 3B). This reversal suggests that the infection‐induced changes in the microbiome are transient and recover over time, despite the age‐associated differences in baseline composition.

On species level, the two most abundant species *Faecalibaculum rodentium* and *Akkermansia muciniphila* are significantly decreased in old mice prior to infection and remained consistently lower throughout the infection, although this difference did not reach statistical significance. *Clostridium* sp. *CAG678*, however, was increased in old mice (Figure 3C). Linear Discriminant Analysis (LDA) calculates scores based on the magnitude of difference in abundance of features between groups; results shown in Figure 3D therefore indicate great effect sizes for the abundance of *Faecalibaculum rodentium* and *Akkermansia muciniphila* in young mice, and for example *Escherichia coli* and *Parabacteroides goldsteinii* in old mice in the acute phase of the infection (Figure 3D). At the species level, samples from old mice showed a more distinct clustering pattern following IAV infection at Day 4 post‐infection, indicating a stronger infection‐associated shift in gut microbiome composition compared with young mice (Figure 3E).

IAV infection altered both the composition and metabolic potential of the gut microbiome, with age‐dependent effects and temporal dynamics. Shotgun metagenomic analysis revealed distinct clustering of CAZy‐annotated genes between young and old mice at Day 4 post‐infection (Figure S5). Notably, enzymes involved in glucose fermentation were transiently upregulated in both age groups relative to mock‐infected controls, with the effect particularly pronounced in old mice. These findings are consistent with the central role of microbial carbohydrate fermentation pathways in SCFA production (Figure 3F,G) (Hackmann 2024; Hornung et al. 2025). LDA scoring highlighted a stronger response in glycosyltransferases (GT) in young mice, which are implicated in bacterial cell wall remodeling and extracellular polysaccharide synthesis (Figure S6).

KEGG‐based annotation indicated a potential decline in pyruvate production via the Entner‐Doudoroff pathway (M00008) and reduced acetate generation through pyruvate dehydrogenase (quinone, EC 1.2.5.1) in old mice (Figure 3H,I), consistent with age‐related metabolic decline.

These findings illustrate the complex interplay between aging, viral infection, and microbiome function. Aging reduces SCFA‐producing species, for instance *Akkermansia muciniphila* , and enriches age‐associated genera such as *Clostridium*. IAV infection transiently elevates glucose fermentation, reflecting a microbiome‐mediated metabolic adaptation, which is less pronounced in older hosts. By the later stages of infection, microbial composition largely returns to baseline, demonstrating resilience despite age‐associated dysbiosis. Together, these results demonstrate that aging is associated not only with compositional changes of the gut microbiota but also with a reduced microbial capacity for SCFA‐associated metabolism during influenza infection. Because acetate represents a major microbiota‐derived metabolite with established immunomodulatory and antiviral properties, these observations prompted us to investigate whether reduced acetate availability directly contributes to the increased susceptibility of senescent lung cells to influenza virus infection.

### 2.4 Acetate Reduces IAV Replication in Senescent Lung Fibroblasts via FFAR2

As we observed that old mice showed a decrease in SCFA‐producing taxa and SCFA‐related metabolic pathways, we tested whether acetate, as a major microbiota‐derived SCFA with known antiviral and immunomodulatory properties, can modulate IAV replication and inflammation. Acetate is linked to FFAR2/FFAR3 signaling and histone acetylation in respiratory virus infection. Viral replication was chosen as the primary readout because it directly reflects cellular permissiveness to IAV. While epithelial cells are the primary targets of IAV replication, lung fibroblasts are key stromal cells that accumulate senescence‐associated changes with aging and contribute to the inflammatory tissue microenvironment. Our previous work showed that senescent lung fibroblasts are permissive to IAV and can amplify inflammatory responses. Therefore, they provide a relevant model to study age‐associated cellular vulnerability and acetate‐mediated modulation in the lung microenvironment. Therefore, we induced senescence in human primary lung fibroblasts (IMR‐90) using doxorubicin, confirmed by upregulation of p21 (*CDKN1A*), downregulated Lamin B1 (*LMNB1*), and elevated parameters of the SASP (Figure 4A,B). Flow cytometry revealed significant overexpression of the SCFA receptors FFAR2 and FFAR3 on senescent cells compared to quiescent controls (Figure 4C).

**FIGURE 4:**
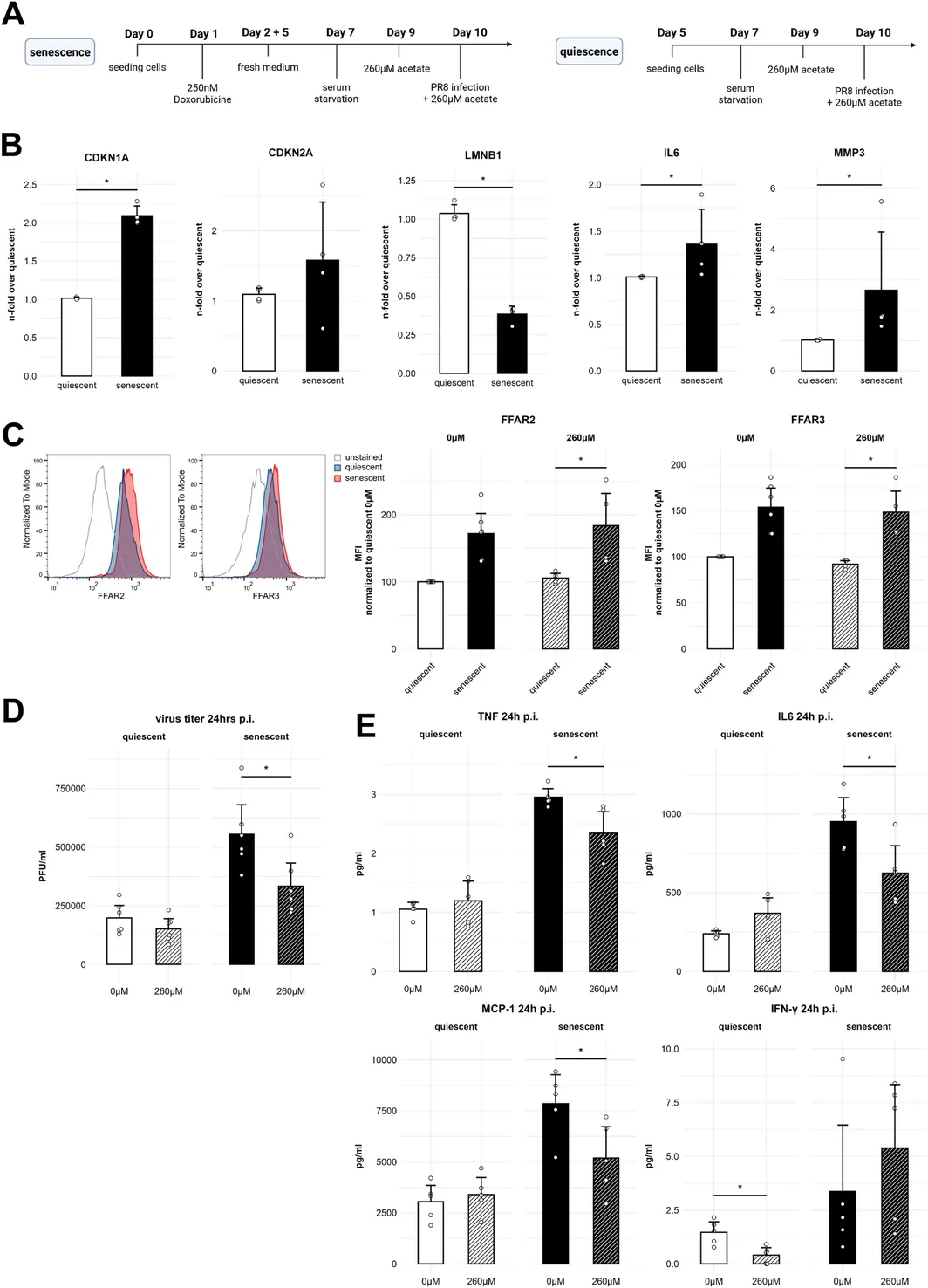
Acetate supplementation reduced influenza A virus (IAV) replication and inflammatory responses in a cellular senescence model using primary human lung fibroblasts (IMR‐90). (A) Schematic representing the workflow of senescence induction, infection with IAV and treatment with acetate. (B) Senescence induction was verified by RT‐qPCR analysis of genes encoding canonical senescence‐associated proteins, including p21, p16, MMP3, lamin B1, and IL‐6. Quiescent cells served as control for senescence (*n* = 4). (C) Expression of free fatty acid receptor 2 and 3 (FFAR2, FFAR3) in senescent and quiescent IMR‐90 cells, quantified as median fluorescent intensity (MFI) via flow cytometry (0 μM acetate: *N* = 6; 260 μM acetate: *N* = 4). (D) Quantification of the virus titer 24 hpi in senescent and quiescent IMR‐90 cells with and without acetate supplementation using plaque assay. (*n* = 6). (E) Cytokine concentrations in cell culture supernatants from senescent and quiescent IMR‐90 cells with and without acetate supplementation at 24 hpi (*n* = 5). *p < 0.05. *p* was calculated by using Mann–Whitney‐Wilcoxon test. ac, acetate; M, mock; Q, quiescent; S, senescent; V, influenza A virus.

Consistent with our prior work, IAV infection in senescent fibroblasts led to markedly higher viral loads than in non‐senescent cells, highlighting their increased susceptibility (Figure 4D). Treatment with various concentrations of acetate alone did not compromise cellular viability or integrity under these conditions (Figure S7). However, pre‐treatment with acetate significantly reduced viral replication and inflammation in senescent cells without affecting viability, indicating a protective effect mediated through the contribution of FFAR2/FFAR3‐associated signaling (Figure 4D,E).

These findings demonstrate that senescent lung fibroblasts are highly permissive to IAV, and that acetate can mitigate viral replication, suggesting a potential therapeutic role for SCFAs in modulating age‐associated cellular vulnerability to respiratory viruses.

### 2.5 Acetate Enhances H3 Acetylation and Suppresses Pro‐Inflammatory Signaling in IAV‐Infected Senescent Fibroblasts

To examine the effects of acetate on senescent lung fibroblasts, we performed RNA sequencing on IAV‐infected IMR‐90 cells. Acetate treatment induced changes to gene expression in both quiescent and senescent fibroblasts (Figure S8). KEGG annotation and enrichment analysis revealed that IAV‐infected senescent cells upregulate numerous pathways related to inflammation and pathogen recognition, including NF‐κB and NOD‐like receptor signaling, whereas these pathways remained largely unchanged in the presence of acetate. Acetate also enhanced pathways related to oxidative phosphorylation and reactive oxygen species production (Figure 5A). IPA (Ingenuity Pathway Analysis) further confirmed that pathogen‐induced cytokine storm signaling was strongly elevated in IAV‐infected senescent cells but was attenuated upon acetate treatment, while pathways linked to cellular senescence were unaffected (Figure 5B). Analysis of cytokine and cytokine receptor expression underscored acetate's modulatory effects on immune responses, revealing age‐associated differences following IAV infection (old IAV vs. young IAV Figure 5C, IAV‐induced changes within each age group are shown in Figure S9).

**FIGURE 5:**
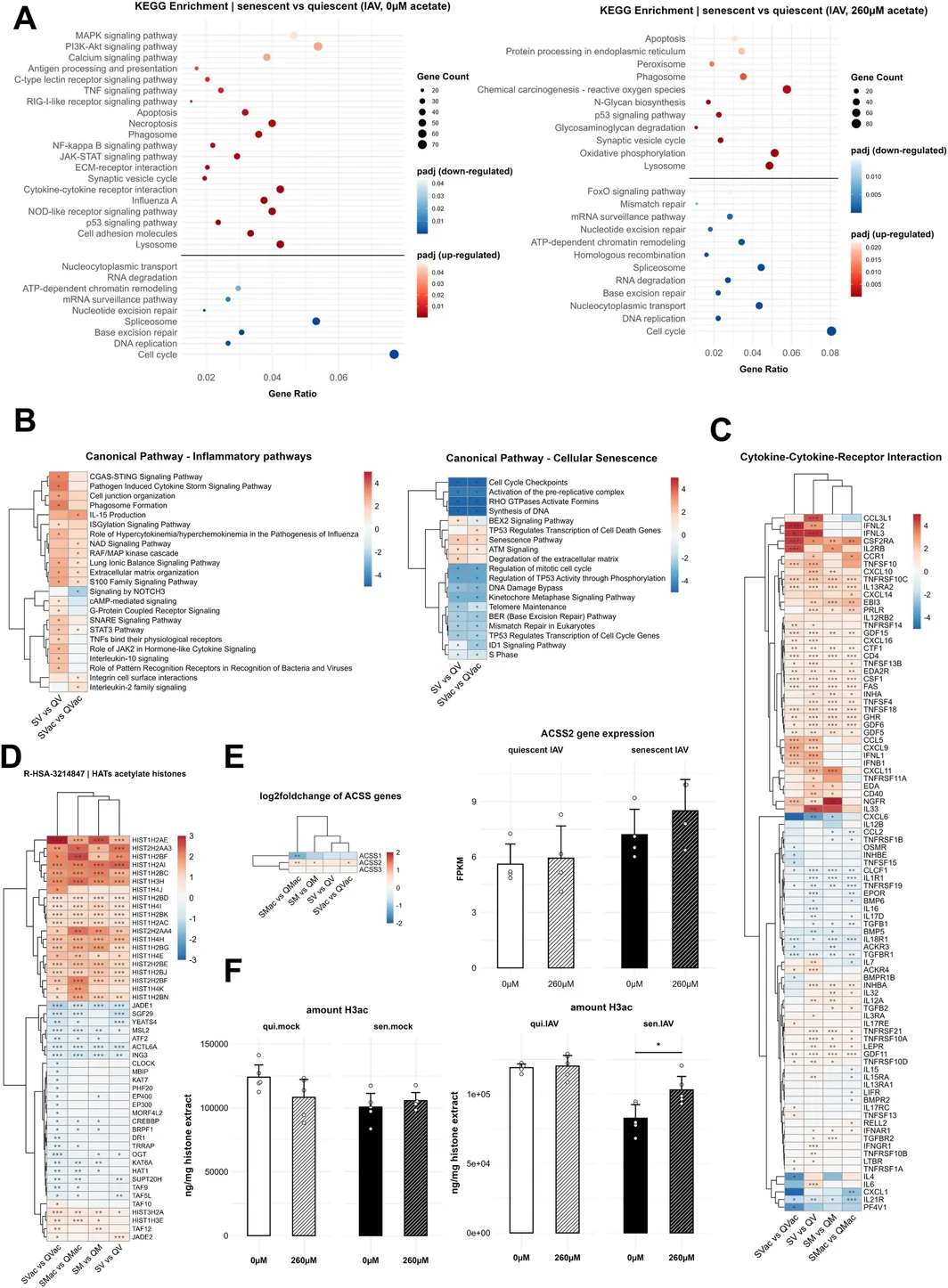
mRNA sequencing analysis of senescent human primary lung fibroblasts (IMR‐90) revealed acetate‐associated changes in gene expression during influenza A virus (IAV) infection (A) KEGG Enrichment analysis comparing up‐ and downregulated pathways in senescent compared to quiescent IMR‐90 cells without acetate supplementation (left) and with supplementation (right). (B) Canonical pathway analysis of cellular senescence using Ingenuity Pathway Analysis (IPA). The heat map displays *z*‐score–based pathway activity for selected senescence‐related canonical pathways across the indicated experimental comparisons, with positive and negative *z*‐scores reflecting predicted pathway activation or inhibition. (C) The impact of senescence on the cytokine‐cytokine interaction during IAV infection and acetate supplementation is demonstrated as gene expression log2foldchange. (D) The impact of senescence on histone acetylation during IAV infection and acetate supplementation is demonstrated as gene expression log2foldchange. The selected genes are based on the Reactome pathway R‐HSA‐3214847. (E) Gene expression log2foldchange (left) and gene expression of *ACSS2*, that encodes a protein relevant in activation of acetate and histone acetylation afterwards during IAV infection and acetate supplementation (F) Quantification of histone H3 acetylation (H3ac) in quiescent and senescent mock‐treated (left) and IAV‐infected IMR‐90 cells exposed to 0 μM or 260 μM compound (*n* = 5). *p < 0.05. *p* calculated by IPA canoncial pathway (B), DESeq2 (C, D, E) and Mann–Whitney‐Wilcoxon test (F).

Given that acetate can serve as a substrate for histone acetylation, we investigated epigenetic modifications in senescent infected cells. Reactome enrichment revealed regulation of R‐HSA‐32148471 (HATs acetylate histones) in both infected and mock‐infected cells, with cellular senescence influencing baseline histone acetylation (Figure 5D). ACSS2, an enzyme activating acetate for histone modification, was upregulated in senescent fibroblasts, supporting its role in chromatin remodeling (Figure 5E). Consistently, histone extracts showed decreased H3 acetylation in IAV‐infected senescent cells, which was restored upon acetate supplementation (Figure 5F). Additionally, senescent cells showed reduced HDAC activity at baseline, which could be restored by supplementation of acetate (Figure S10).

Together, these results demonstrate that acetate reshapes transcriptional networks in senescent fibroblasts, downregulates inflammatory pathways, modulates antiviral gene expression, and enhances H3 acetylation. This epigenetic remodeling likely underlies the protective effects of acetate, reducing cytokine overproduction and viral replication in IAV‐infected old cells.

## 3 Discussion

Our study demonstrates that aging‐associated alterations of the gut microbiota are accompanied by a reduced capacity for SCFA production and impaired antiviral defense during IAV infection. By combining a murine model of aging and influenza infection with mechanistic experiments in human senescent lung fibroblasts, we provide evidence linking age‐associated microbial dysfunction with impaired cellular antiviral responses. These findings offer a translational framework that may help explain the increased severity of influenza observed in older individuals. Aging is a major risk factor for respiratory infections, particularly IAV, with individuals over 60 years experiencing higher morbidity, mortality, and prolonged hospitalization (Miron et al. 2024).

Our in vivo findings demonstrate a clear age‐dependent response to IAV infection in C57BL/6 mice. Rather than mounting a rapid and transient immune response, old mice exhibited delayed viral clearance accompanied by prolonged inflammatory activation, suggesting that aging compromises the coordination between early antiviral immunity and subsequent resolution of inflammation. These data are consistent with age‐associated immune dysregulation during influenza infection, while extending these findings by linking impaired antiviral defense to concurrent alterations in gut microbial metabolism (Hernandez‐Vargas et al. 2014).

Beyond confirming age‐associated changes in microbiota composition, our analyses identified alterations in microbial metabolic pathways related to SCFA production. IAV infection transiently increased *Akkermansia* abundance and glucose fermentation pathways during the acute phase, suggesting a potential microbiome‐mediated adaptation to infection (De Siqueira et al. 2023; Hackmann 2024). This observation is particularly relevant because microbial metabolites rather than taxonomic composition are increasingly recognized as functional mediators of host immunity. The reduced abundance of SCFA‐producing taxa together with diminished acetate‐associated metabolic pathways therefore suggested a mechanistic link between gut microbiota dysfunction and impaired antiviral defense during aging.

Importantly, these in vivo observations generated the biological hypothesis that reduced microbiota‐derived acetate contributes directly to increased susceptibility of senescent lung cells to influenza virus infection. This hypothesis formed the rationale for the subsequent mechanistic experiments in our human in vitro model.

To investigate whether the age‐associated reduction in SCFA‐related metabolic capacity observed in vivo has functional consequences for antiviral defense, we employed a complementary in vitro human senescent lung fibroblast model. In line with our previous findings, senescent fibroblasts exhibited increased susceptibility to IAV infection, supporting the concept that aging‐associated cellular alterations promote viral replication within the lung microenvironment (Schulz et al. 2023). Importantly, our study demonstrates for the first time that acetate restores antiviral defense in senescent human lung fibroblasts. Acetate supplementation significantly reduced viral replication and dampened inflammatory responses, thereby identifying microbiota‐derived acetate as a functional mediator linking age‐associated microbial dysbiosis with impaired antiviral immunity. These findings considerably extend the observations from the murine model by providing mechanistic evidence that reduced microbial metabolite availability is not merely associated with aging but directly influences the susceptibility of senescent lung cells to influenza virus infection.

The observed effects are consistent with modulation of metabolic and epigenetic pathways and support an emerging concept in which microbiota‐derived metabolites actively shape antiviral immunity in aging tissues (Antunes et al. 2022; Niu et al. 2023).

Our results further reveal that senescent fibroblasts express higher levels of FFAR2 and FFAR3, receptors known to mediate SCFA signaling, supporting an increased responsiveness to microbiota‐derived metabolites in the aging lung (Antunes et al. 2019; Xu et al. 2019). Corroborating our previous work, acetate promoted receptor internalization, providing a plausible explanation for the reduced viral replication observed following acetate supplementation (Hornung et al. 2025). Beyond its effects on viral entry, acetate profoundly altered the host response of senescent cells. Transcriptomic analyses of acetate‐treated senescent cells identified downregulation of inflammation‐related pathways, while pathways associated with oxidative phosphorylation were enhanced, suggesting that acetate promotes a cellular state that is less permissive to influenza virus replication while simultaneously limiting excessive inflammatory activation.

A particularly important finding of our study is that acetate restored the reduction in histone H3 acetylation observed following IAV infection in senescent fibroblasts. Rather than representing an isolated epigenetic observation, this finding provides a potential mechanistic explanation for the broad transcriptional changes induced by acetate. The simultaneous restoration of H3 acetylation, attenuation of inflammatory signaling, and reduction of viral replication suggests that microbiota‐derived acetate influences antiviral immunity through coordinated metabolic and epigenetic reprogramming.

Previous studies have demonstrated that acetate can serve as a substrate for histone acetylation and thereby modulate gene expression (Bolduc et al. 2017; Mews et al. 2019). Our data extend these observations by demonstrating that acetate‐mediated epigenetic remodeling occurs in the context of influenza infection and senescent human lung cells, thereby providing a potential mechanistic link between age‐associated microbial dysbiosis and impaired antiviral defense.

Collectively, our findings suggest that the functional consequences of age‐associated gut microbiota dysbiosis are mediated not only through altered microbial composition but also through changes in microbial metabolite availability that directly reprogram antiviral responses in senescent lung cells.

While this experimental design enabled us to identify age‐associated alterations in microbial metabolism and subsequently investigate their functional consequences in human senescent lung cells, future studies integrating direct SCFA quantification, metabolomic profiling of primary old human lung tissue will be required to establish a causal relationship between microbiota‐derived metabolites and antiviral defense during aging. Ultimately, validation in clinical cohorts will be important to determine whether circulating or microbiota‐derived acetate levels correlate with influenza severity and antiviral resilience in older individuals.

By integrating observations from a combined murine model of aging and influenza infection with mechanistic validation in human senescent lung cells, our study provides a mechanistic framework linking the aging gut microbiome to impaired antiviral immunity and identifies microbiota‐derived acetate as a promising target for future host‐directed antiviral therapies.

## 4 Materials and Methods

### 4.1 Virus Strains and Propagation

In vivo infections were conducted with H1N1 influenza A virus strain HA‐G22‐mpJena/5258 which was isolated as previously published (Seidel et al. 2014). The murine in vivo experiments used this mouse‐adapted IAV strain, as standard for robust and reproducible infection in C57BL/6 mice. This strain has been used in several previous virological studies from our group (Hornung et al. 2025; Reisser et al. 2024; Schulz et al. 2023). For in vitro infections the influenza strain H1N1 influenza A virus/Puerto Rico/8/34 (PR8) was used. For the in vitro experiments, PR8 was used because it is a well‐established laboratory H1N1 strain for infection studies in cultured human cells and allows comparison with previous mechanistic work.

Viruses were propagated on Madin‐Darby canine kidney (MDCK) cells cultured in minimal essential medium (MEM; Thermo Fisher Scientific, Waltham, USA) containing 1% penicillin/streptomycin (P/S, Lonza, Basel, Switzerland) and 10% Fetal bovine serum (FBS; PAN‐Biotech GmbH, Aidenbach, Germany).

### 4.2 In Vivo Infection Mouse Model and Sample Collection

Approval for murine in vivo experiments was obtained from the Thuringian State Office for Consumer Protection (TV‐Number: UKJ‐22‐023).

Female C57BL/6 mice, either young (8–12 weeks; *n* = 48) or old (18–24 months; *n* = 48), were purchased from Janvier Labs (Le Genest‐Saint‐Isle, France). Female C57BL/6 mice were used to maintain consistency with established IAV infection protocols in our laboratory and previous studies using this model, thereby reducing biological variability and allowing comparison with prior datasets. Mice were divided into mock and IAV infection groups. Murine studies were neither randomized nor blinded.

Before infection, mice were anesthetized with 2% isoflurane and intranasally infected with 10^6^ plaque‐forming units (PFU) of HA‐G22‐mpJena/5258 in 20 μL PBS (10 μL per nostril). Weight and clinical burden scores were assessed daily for up to 21 days, as previously described (Hornung et al. 2025) (Table S1). Age‐related additions to the clinical scoring system included slight and moderate lethargy, as well as changes in activity or posture, which are established clinical signs used to monitor influenza morbidity in older mice (Bartley et al. 2016; Toapanta and Ross 2009). In old mice, influenza infection is associated with altered sickness behavior, often more prolonged or emphasized, supporting the inclusion of these readouts as sensitive indicators of disease severity in high‐risk cohorts (Bartley et al. 2016). Mice were sacrificed 4, 8, and 21 days post‐infection using ketamine 500 mg/kg body weight and xylazine 80 mg/kg body weight (i.p.), corresponding to a fivefold overdose, with a maximum injection volume of 10 mL/kg body weight. A total of 96 mice were included in the experiment. At each planned time point (4, 8, and 21 dpi), each mock‐infected group comprised 6 young and 6 old mice, whereas each IAV‐infected group comprised 10 young and 10 old mice. Of the mice designated for sacrifice on Day 21, 4 of 10 IAV‐infected young mice and 7 of 10 IAV‐infected old mice reached this time point. The remaining infected mice met humane endpoint criteria before Day 21. All mock‐infected mice in both age groups reached day 21.

Bronchoalveolar lavage (BAL) was collected by flushing the lungs with PBS. The right inferior lung lobe was homogenized in Minimal Essential Medium (MEM, Sigma Aldrich, Taufkirchen, Germany) normalized to tissue weight. Viral load was then quantified via plaque assay in both lung tissue and BAL samples. The right superior lung lobe was homogenized in RLT buffer for RNA extraction. Blood was collected, coagulated at room temperature, centrifuged at 5000 × *g* for 10 min, and serum was harvested. Cytokine levels were measured in BAL and serum samples. Fecal samples were collected from 0, 4, and 21 d post‐infection for metagenomic shotgun sequencing.

### 4.3 DNA Extraction From Feces and Shotgun‐Metagenomic Analysis

DNA was extracted by use of the QIAmp PowerFecal Pro DNA Kit (Qiagen, Hilden, Germany) as stated in the instruction manual. Metagenomic sequencing was performed by Novogene UK. Genomic DNA was randomly sheared, end‐repaired, and A‐tailed, followed by ligation with Illumina platforms. Libraries were PCR‐amplified, size‐selected, purified, pooled, and sequenced on Illumina platforms. Novogene performed bioinformatic analysis, including quality control, metagenome assembly, and gene prediction with MetaGeneMark. Taxonomic annotation was based on the microNR database, while functional profiles were generated using KEGG and CAZy. Group comparisons were conducted with Metastat to assess taxonomic and functional differences. All raw sequencing data from metagenomic analysis were made available in the NCBI Sequence Read Archive under the BioProject accession number PRJNA1376233. The data are currently available via a SRA review link (https://dataview.ncbi.nlm.nih.gov/object/PRJNA1376233?reviewer=copt5cqof298b6jfh2odtiqrsa) and will be released upon publication.

### 4.4 RNA Extraction, cDNA Synthesis, and RT‐qPCR

Tissue from murine lungs and cells from human in vitro experiments with IRM‐90 cells were lysed in RLT buffer. RNA was isolated using the Qiagen RNAeasy Mini Kit (Qiagen, Hilden, Germany) according to the manufacturer's instructions. RNA concentrations were measured using a NanoDrop spectrophotometer ND‐1000 (Peqlab/VWR, Radnor, USA). Reverse transcription was applied to synthesize cDNA from RNA of human IMR‐90 cells by using the High‐Capacity cDNA Reverse Transcription Kit (Thermo Fisher Scientific, Waltham, USA). Real‐time quantitative PCR (RT‐qPCR) (95°C for 10 min, followed by 45 cycles of 95°C for 10 s, 60°C for 20 s, and 72°C for 30 s) was performed with the Maxima SYBR Green qPCR Master Mix (Thermo Fisher Scientific, Waltham, USA) and Rotor‐gene Q (Qiagen). The expression of genes of interest was quantified using the 2^−ΔΔCT^ method with the expression of the β‐actin gene as a reference gene for normalization as it was stably expressed in the analyzed human cell culture conditions under our experimental settings. Primer sequences for genes are listed in Table S2.

### 4.5 mRNA Sequencing

For sequencing of mRNA from murine lung tissue and human IMR‐90 cells, RNA samples were processed by Novogene Co. LTD (Beijing, China) using the Illumina platform NovaSeq 6000 S4 flowcell v1.0, based on the mechanism of sequencing by synthesis (SBS) and the PE150 strategy. In the subsequent bioinformatic analysis, Raw FASTQ reads were initially processed using fastp. Reads with adapter sequences, poly N regions, and low quality were removed to generate clean data. The clean reads were then aligned to the *Mus musculus* reference genome (GRCm39/mm39) or the *Homo sapiens* reference genome (GRCh38.p12) respectively using HISAT2 software. Gene expression levels were quantified employing the FPKM (fragments per kilobase per million) method. Differential gene expression was determined with DESeq2 software, utilizing a negative binomial distribution. The Benjamini‐Hochberg procedure was applied to calculate the false discovery rate (FDR). Analysis of functional pathways was performed by matching sequencing data with KEGG and Reactome databases, as well as QIAGEN Ingenuity Pathway Analysis (IPA). Gene expression data were also submitted to NCBI Gene Expression Omnibus under accession number GSE313182. As with SRA, data at GEO can be accessed with the following reviewer token (anqzgummxvopdot) and will be made public upon publication. Further data derived from these datasets such as DEG files were uploaded at DOI (Mendeley Data, V1, doi:10.17632/9pw3yyyr4c.1, https://data.mendeley.com/preview/9pw3yyyr4c?a=529d7ba3‐1ccb‐4164‐9ae1‐0b7fa406d680).

Heat maps to display the results of differentially expressed genes (DEG) analysis or IPA were created by using R (v4.2.2; R Foundation for Statistical Computing, https://www.r‐project.org/). The log2 fold change and gene ratio of IPA canonical pathways are displayed respectively. Significant differences in expression were emphasized by marking respective cells with asterisks (*p ≤ 0.05; **p ≤ 0.01; ***p ≤ 0.001).

### 4.6 Cell Culture

For in vitro experiments, primary human lung fibroblasts (IMR‐90 cells, Coriell Institute for Medical Research, Camden, USA) were used. These cells were cultured in Dulbecco's modified Eagle's medium (DMEM, Sigma Aldrich, Taufkirchen, Germany) supplemented with 10% fetal bovine serum (FBS, PAN Biotech, Aidenbach, Germany) and 1% penicillin/streptomycin (P/S, Lonza, Basel, Switzerland).

Further analysis utilized MDCK cells. These were kept in cell culture with MEM, supplemented with 10% FBS and 1% P/S.

### 4.7 Induction of Cellular Senescence

Cellular senescence in IMR‐90 cells was induced with doxorubicin‐hydrochloride (2 mg/mL stock, Sigma‐Aldrich) diluted to 250 nM in culture medium. Cells were treated for 24 h, after which the medium was replaced with fresh DMEM (10% FBS, 1% P/S). A second medium change was performed 2 days later. Quiescent cells served as control cells to the Doxorubicin‐treated cells. To achieve this state of reversible replicative arrest, cells underwent serum starvation by reduction of serum concentration to 0.2% FBS for 2 days, and then to 0% FBS for another 24 h. This step of serum starvation was also performed on the cells previously treated with doxorubicin to minimize artificial differences between those two groups. 10 days after doxorubicin treatment, the effectiveness of treatment was tested utilizing senescence‐associated β‐galactosidase staining (SA β‐gal staining, Biovision, Cambridge, UK), according to the manufacturer's instructions.

### 4.8 Stimulation With Acetate

For treatment of IMR‐90 human lung fibroblasts with acetate, a concentration of 260 μM was chosen as described in previous studies (Antunes et al. 2019). Sodium acetate (≥ 99%, anhydrous, Roth, Karlsruhe, Germany) was prepared in endotoxin‐free water, sterile‐filtered, and diluted to 260 μM in DMEM (10% FBS, 1% P/S). Treatment was applied for 24 h prior to infection and maintained until sample collection (8 or 24 h post‐infection).

### 4.9 Quantification of Cytotoxicity

Cytotoxicity following acetate treatment was assessed in cell culture supernatants from senescent and quiescent IMR‐90 fibroblasts using the CyQUANT LDH Cytotoxicity Assay (Thermo Fisher Scientific, Waltham, MA, USA).

### 4.10 In Vitro Infections

Infection of the in vitro model was performed using influenza A virus/IAV/H1N1/Puerto Rico/8/1934 (PR8). After induction of cellular senescence and stimulation with 260 μM acetate, senescent and quiescent cells were infected with the IAV strain PR8 at a multiplicity of infection (MOI) of 1.

Cells were washed with Dulbecco's phosphate‐buffered saline (DPBS, Thermo Fisher Scientific, Waltham, USA) and incubated for 30 min at 37°C, 5% CO_2_ with virus diluted in DPBS containing 0.2% bovine serum albumin ((BSA, Roth, Karlsruhe, Germany), 1 mM MgCl_2_ (Sigma Aldrich) and 0.9 mM CaCl_2_). Following this, virus‐containing DPBS was discarded and DMEM with 0.2% BSA, 1 mM MgCl_2_, 0.9 mM CaCl_2_ and 30 ng/mL TPCK‐treated trypsin was added onto the cells for 24 h. Supernatants from infected and mock‐infected cells were collected to analyze virus replication using a standard plaque assay that was performed as described elsewhere (Deinhardt‐Emmer et al. 2021).

### 4.11 Immunofluorescence Staining of Murine Lung Tissue

The murine left lung lobes were frozen in Tissue‐Tek (Sakura) and cut into 10 μm slices on a Leica CM1950 Cryostat (Leica). Slices were fixed in 4% PFA for 15 min, washed 3 times in PBS, and stained with Alexa Fluor Plus 647 Phalloidin (Invitrogen, Cat# A30107) diluted 1:400 in Dako Antibody Diluent (Dako) for 1 h at room temperature (RT). Coverslips were mounted using DAPI Fluoromount‐G (Southern Biotech), and imaging was conducted using a BZ‐X810 Fluorescence Microscope (Keyence). Images were analyzed with the BZ‐X800 Analyzer (v.1.1.2; Keyence).

### 4.12 Flow Cytometry Analysis of IMR‐90 Human Lung Fibroblasts

Quantification of FFAR2 and FFAR3 was performed via flow cytometry. IMR‐90 cells were detached with accutase (Sigma Aldrich) for 15 min, washed with PBS and stained with Fixable Viability Stain 780 (BD Bioscience) for 15 min at RT. Cells were then washed with PBS, fixed and permeabilized with Fixation/Permeabilization Solution (BD Bioscience) for 20 min on ice. Cells were washed with Fix/Perm Wash Buffer (BD Bioscience) and stained with Human FFAR2/GPR43 Alexa Fluor 647‐ (1:100; R&D Systems, Cat#FAB10082R) and Human FFAR3/GPR41 Alexa Fluor 488‐conjugated Antibody (1:100; R&D Systems, Cat#FAB10562G) for 30 min on ice. After washing with Fix/Perm Wash Buffer, cells were measured on a FACSymphony A1 (BD Bioscience) and analyzed via FloJo (v10.10).

Quantification of cytokine concentrations was performed via the multiplexed immunoassay LEGENDplex Mouse‐ or Human Anti‐Virus Response Panel (Biolegend) according to the manufacturer's instructions. Samples were quantified on a FACSymphony A1 (BD Bioscience) and analyzed via LEGENDplex Data Analysis Software (Qognit, v2024‐06‐15).

### 4.13 Histone Extraction and Histone Acetylation Measurement

For the extraction of histones from senescent and quiescent human IMR‐90 fibroblasts under mock, IAV‐infected, and acetate‐treated conditions, cells were first detached from the well surface by using TPCK‐treated trypsin and afterwards treated with the Histone Extraction Kit (ab113476; Abcam, Cambridge, UK) according to the manufacturer's instructions. After extraction, the concentration of protein extracts was determined via BCA assay using the Pierce BCA Protein Assay Kit (Thermo Fisher Scientific). Extracts were then analyzed with the H3 Acetylation Detection Fast Fluorometric Kit (ab131561, Abcam, Cambridge, UK) according to the instruction manual.

### 4.14 Extraction of Nuclear Protein Fraction and Measurement of Histone Deacetylase Activity

Similar to histone analysis, after detachment of fibroblasts, the nuclear protein fraction containing the histone deacetylases relevant in epigenetic modification was extracted utilizing the NE‐PER Nuclear and Cytoplasmic Extraction Reagents (Thermo Fisher Scientific, Waltham, USA) and HDAC activity was measured with the Histon‐Deacetylase (HDAC) activity assay kit (fluorometric) (Thermo Fisher Scientific, Waltham, USA), both according to protocol.

### 4.15 Statistical Analysis and Illustrations

Data is displayed in bar plots as mean with error bars ± SD. Statistically significant differences are indicated by asterisks. Calculation of *p*‐values was performed utilizing Two‐way ANOVA and Wilcoxon signed‐rank test (*p ≤ 0.05; **p ≤ 0.01; ***p ≤ 0.001). Statistical analysis as well as creation of graphics was done using GraphPad Prism v9.0 and R (v4.2.2).

Graphical abstract as well as study designs were designed with Biorender.com.

## Author Contributions

Conceptualization: S.D.‐E.; methodology: C.W., F.H., A.H., Y.R., C.J.L.S, K.G.; writing – original draft preparation: S.D.‐E., C.W.; writing – review and editing, all authors; visualization: S.D.‐E., C.W.; resources and supervision: S.D.‐E.; funding acquisition, S.D.‐E. All authors have read and agreed to the published version of the manuscript.

## Funding

This work is supported by the BMFTR, funding program Photonics Research Germany (“LPI‐BT1‐FSU,” FKZ 13N15466; “LPI‐BT2‐IPHT,” FKZ 13N15704, and “SARS‐CoV‐2Dx,” FKZ: 13N15745) and is integrated into the Leibniz Center for Photonics in Infection Research (LPI). The LPI initiated by Leibniz‐IPHT, Leibniz‐HKI, UKJ, and FSU Jena is part of the BMFTR national roadmap for research infrastructures. In addition, this work was supported by funding from the Foundation “Else Kröner‐Fresenius‐Stiftung” within the Else Kröner Graduate School for Medical Students “Jena School for Aging Medicine (JSAM).”

## Ethics Statement

The study was approved by the local ethics committee of the Jena University Hospital (no. 2018‐1263, 2020‐1894, and no. 2020‐1773).

## Consent

The authors have nothing to report.

## Conflicts of Interest

The authors declare no conflicts of interest.

## Supporting information


**Figure S1:** Body weights (expressed as % change from day 0) of influenza A virus (IAV) infected young and old mice were measured daily for 21 days. A total of 30 young and 30 old IAV infected mice were included, comprising 10 animals per age group and time point (Days 4, 8, and 21 post infection). **p < 0.01, ****p < 0.0001. *p* calculated utilizing 2way ANOVA Šídák's multiple comparisons test.
**Figure S2:** Concentration of cytokines measured in BAL of uninfected mice. Cytokine quantification was performed in the BAL of young and old mice using flow cytometry. **p < 0.01, ***p < 0.001. *p* calculated using Mann–Whitney‐Wilcoxon test (*n* = 16 independent replicates).
**Figure S3:** Influenza A virus (IAV) induced inflammatory changes in the lung tissue of young and old mice. Heat maps show log2 fold changes in the expression of inflammation‐related genes derived from mRNA sequencing of lung tissue. Young (left) and old (right) IAV‐infected mice were compared with their respective age‐matched mock‐treated controls at 4, 8, and 21 dpi. y = young; o = old. *p < 0.05.
**Figure S4:** Influenza A virus (IAV) induced changes in the lung tissue of young and old mice. Reactome pathway enrichment analysis of lung tissue transcriptomes from young (A) and old (B) IAV‐infected mice at 8 dpi, each compared with their respective age‐matched mock‐treated controls.
**Figure S5:** Influenza A virus (IAV) infection resulted in a shift in the microbial composition of the gut. Principal Component Analysis of genes annotated to CAZy database (EC level). Samples are grouped by age, treatment, and sampling time point. IAV, influenza A virus; M, mock; O, old; Y, young.
**Figure S6:** Changes in glycosyltransferase (GT) families suggest altered carbohydrate metabolism and fermentative activity in the gut microbiome. Linear discriminant analysis (LDA) of CAZy Level 2 families in gut microbiota samples collected at 4 dpi. Bars represent LDA scores (log10) for differentially abundant carbohydrate‐active enzyme families across the indicated groups. CBM, carbohydrate‐binding module; GH, glycoside hydrolase; GT, glycosyltransferase; IAV, influenza A virus; O, old; Y, young.
**Figure S7:** Cell number and cytotoxicity of lung fibroblasts (IMR‐90 cells) cultured in the presence or absence of 260 μM acetate. Cell number was assessed by cell counting, and cytotoxicity was determined using an LDH assay (*n* = 3 independent replicates).
**Figure S8:** Venn diagrams showing differentially expressed genes in quiescent and senescent influenza A virus (IAV)‐infected human primary lung fibroblasts (IMR‐90) following acetate supplementation. Quiescent and senescent IMR‐90 cells were treated with 260 μM acetate for 24 h and subsequently either mock‐treated or infected with IAV for 24 h. Gene expression profiles were obtained by mRNA sequencing. Acetate supplementation altered gene expression patterns in both quiescent and senescent IMR‐90 cells under mock‐treated and IAV‐infected conditions. (A) Effect of acetate and IAV on quiescent or senescent IMR‐90 cells. (B) Effect of acetate and the quiescent and senescent phenotype in mock‐treated or IAV‐infected IMR‐90 cells. Ac, acetate; iav, influenza A virus; Q, quiescent; S, senescent.
**Figure S9:** Senescent human primary lung fibroblasts (IMR‐90) revealed acetate‐associated changes in expression of cytokine‐associated genes during influenza A virus (IAV) infection. Heat maps show log2 fold changes in the expression of cytokine‐ and cytokine receptor–associated genes in senescent and quiescent human primary lung fibroblasts (IMR‐90) following IAV infection. IAV‐infected cells were compared with their respective mock‐treated controls, with and without acetate supplementation. ac, acetate; M, mock; Q, quiescent; S, senescent; V, influenza A virus. *p < 0.05, **p < 0.01, ***p < 0.001.
**Figure S10:** Histone deacetylase (HDAC) activity was assessed in mock‐ and influenza A virus (IAV)‐infected quiescent and senescent IMR‐90 fibroblasts with and without acetate supplementation. HDAC activity was measured with the HDAC activity assay in the nuclear protein fraction of quiescent (left) and senescent (right) IMR‐90 cells. **p < 0.01. *p* calculated utilizing Mann–Whitney‐Wilcoxon test. *n* = 5 independent replicates. qui, quiescent; sen, senescent.
**Table S1:** Clinical scoring criteria used for daily health assessment of influenza A virus (IAV) infected young and old mice. Individual symptom scores were recorded daily from Day 0 to Day 21 post infection and summed for each animal; animals reaching a cumulative daily score of ≥ 25 were considered to have met humane endpoint criteria. Selected clinical parameters were weighted accordingly to capture respiratory infection severity in combination with age‐associated differences in baseline.
**Table S2:** Forward and reverse sequence of primers used in PCR analysis.

## Data Availability

All raw sequencing data from metagenomic analysis and RNA‐seq was made available in the NCBI Sequence Read Archive under the BioProject accession number PRJNA1376233. The data are currently available via a SRA review link (https://dataview.ncbi.nlm.nih.gov/object/PRJNA1376233?reviewer=copt5cqof298b6jfh2odtiqrsa) and will be released upon publication. Gene expression data were also submitted to NCBI Gene Expression Omnibus under accession number GSE313182. As with SRA, data at GEO can be accessed with the following reviewer token (anqzgummxvopdot) and will be made public upon publication. Further data derived from these datasets such as DEG files were uploaded at DOI (Mendeley Data, V1, doi:10.17632/9pw3yyyr4c.1, https://data.mendeley.com/preview/9pw3yyyr4c?a=529d7ba3‐1ccb‐4164‐9ae1‐0b7fa406d680). For statistical analysis and illustration of results no special codes were used.
